# Circular RNA Profiles in Viremia and ART Suppression Predict Competing circRNA–miRNA–mRNA Networks Exclusive to HIV-1 Viremic Patients

**DOI:** 10.3390/v14040683

**Published:** 2022-03-25

**Authors:** Dora Zucko, Abdullgadir Hayir, Kelsey Grinde, Kathleen Boris-Lawrie

**Affiliations:** 1 Department of Veterinary and Biomedical Sciences, University of Minnesota, Saint Paul, MN 55108, USA; zucko001@umn.edu (D.Z.); ahayir@macalester.edu (A.H.); 2 Department of Mathematics, Statistics and Computer Science, Macalester College, Saint Paul, MN 55105, USA; kgrinde@macalester.edu

**Keywords:** circular RNAs, microRNA response elements, competing endogenous RNA networks, RNA silencing suppressor, post-transcriptional regulation, bioinformatics

## Abstract

Since the onset of the HIV-1/AIDS epidemic in 1981, 75 million people have been infected with the virus, and the disease remains a public health crisis worldwide. Circular RNAs (circRNAs) are derived from excised exons and introns during backsplicing, a form of alternative splicing. The relevance of unconventional, non-capped, and non-poly(A) transcripts to transcriptomics studies remains to be routinely investigated. Knowledge gaps to be filled are the interface between host-encoded circRNAs and viral replication in chronically progressed patients and upon treatment with antiviral drugs. We implemented a bioinformatic pipeline and repurpose publicly archived RNA sequence reads from the blood of 19 HIV-1-positive patients that previously compared transcriptomes during viremia and viremia suppression by antiretroviral therapy (ART). The in silico analysis identified viremic patients’ circRNA that became undetectable after ART. The circRNAs originated from a subset of host genes enriched in the HDAC biological pathway. These circRNAs and parental mRNAs held in common a small collection of miRNA response elements (MREs), some of which were present in HIV-1 mRNAs. The function of the MRE-containing target mRNA enriched the RNA polymerase II GO pathway. To visualize the interplay between individual circRNA–miRNA–target mRNA, important for HIV-1 and potentially other diseases, an Interactive Circos tool was developed to efficiently parse the intricately competing endogenous network of circRNA–miRNA–mRNA interactions originating from seven circRNA singled out in viremic versus non-viremic patients. The combined downregulation of the identified circRNAs warrants investigation as a novel antiviral targeting strategy.

## 1. Introduction

Circular RNAs (circRNAs) are generated during backsplicing, a form of alternative splicing [1]. Genes expressing circRNAs are less efficiently spliced compared with control genes and are more likely to modulate alternative splicing through RNA splicing regulatory sequences [2]. CircRNA are derived from excised exons and introns whose ends are ligated and therefore lack 5’ and 3’ terminal structures [1,3]. Looping interactions between complementary sequences induce circularization of parental pre-mRNA and promote backsplicing. Whereas mature alternatively spliced mRNAs serve as templates for protein translation, circRNA serve as depots for storage of complementary non-coding RNAs and RNA-binding proteins required for post-transcriptional control of gene expression in human and animal species [1]. One of the most widely studied roles for circRNAs is serving as MRE that sequester miRNAs to regulate post-transcriptional gene expression [4,5,6,7].

Backsplicing is a co-transcriptional process that is competitive with canonical splicing [2]. Recent evidence has shown that upon infection by RNA virus, the alternative splicing landscape of host cells is affected [8]. The HIV-1 accessory protein, Vpr, mediates inhibition of the spliceosome, while Tat was shown to modulate splicing of tau [8]. Moreover, a perturbed balance between transcription and linear splicing creates important preconditions for upregulating circRNA biogenesis when active spliceosomes are limiting [9]. By example, cells infected with hepatitis C virus (HCV) showed infection-induced abundance in circRNA population that was not followed by their linear counterparts, an observation that could be explained by virus-induced aberrations in the usage of active splice sites [10]. Hence, viral upregulation of circRNA has the potential to negatively regulate host mRNAs to influence viral replication.

Antagonism of RNA–mRNA networks is a hallmark of viral pathogens infecting hosts within the plant and animal kingdoms and has been attributed to viral RNA silencing suppressor (RSS) activity [11]. An example is the HIV-1 Tat protein, which suppresses Dicer processing of dsRNA [11,12]. One study has also explored the possibility of RNAs to act as RSS and discovered that HIV-1 TAR and RRE RNAs recapitulate RSS activity by competing with small interfering RNAs for incorporation into RISC [13]. We postulated that circRNA have the capacity to be host-derived nucleic acid RSS, which may be exploited by HIV-1 and other viruses in hosts.

Physiological roles for circRNAs have been assigned to infection, most recently to SARS-CoV2 [14,15], and also in neoplastic disease states that occur in a diversity of species (reviewed in [1,16,17,18]). For instance, circRNA antagonism of miRNA activity has shown to inhibit tumor suppressor genes or to inappropriately activate oncogenes through complementary binding of miRNAs at a 6-8 nt seed sequence [2,19]. The miRNAs response elements (MREs) located in 3’ UTRs of mRNAs bound by miRNAs negatively regulate the cognate mRNA. By serving as MRE mimics, circRNA sequester complementary miRNAs and positively regulate post-transcriptional expression of cognate MRE-containing mRNAs [20,21]. Expression profiles of mRNAs and miRNAs in people living with HIV-1 have been used to predict host mRNA–miRNA interacting networks [22,23]. However, we identified only a single study investigating the significance of circ-RNA–miRNA–mRNA interacting networks in the context of retrovirus infection [23]. 

Zhang et al. identified circRNAs in the PBMC of three people in the early stages of HIV-1 infection (<180 days since diagnosis) naive to antiretroviral therapy (ART) and three healthy controls by Illumina sequencing of cDNA libraries generated without selection of poly(A) RNAs [23]. The study results also identified 1365 circRNAs to be differentially expressed in HIV-1 patients and healthy controls and predicted circRNA had roles in HIV-1 replication by regulating the expression of CCNK, CDKN1A, and IL-15. Thirty MREs were identified in common between the upregulated circRNA and the downregulated mRNA, providing rationale for further investigation of circRNA profiles in larger cohorts of people living with HIV-1. In this study, we scrutinized circRNA profiles in the PBMC collected in pre- and post-ART from 19 individuals after Illumina sequencing results had been generated without selection of poly(A) RNAs. As described in [1], the processing of an RNA sample to enrich circRNA molecules avoids selection for polyadenylated mRNA and employs random hexamers to prime cDNA synthesis. Given these caveats, published transcriptomics data [24] deposited in public sequence-read archive repositories were proposed to serve in identifying circRNA profiles useful to predict circRNA–miRNA–mRNA networks influencing viral replication.

## 2. Materials and Methods

### 2.1. Processing of Sequencing Data of Circulating CD4+ T Lymphocytes Isolated from Blood of 19 Patients

In El-Diwany et al. (2017) [25], whole blood was collected from 19 individuals with HIV-1/HCV coinfection having received <24 months of ART over their entire lives and none within 6 months of enrollment (designated pre-ART). Viremia was defined by HIV-1 RNA level > 400 c/mL and HCV RNA > 100,000 IU/mL for >6 months. In addition, whole blood was collected from the same individuals after ART treatment (raltegravir, tenofovir disoproxil fumarate, and emtricitabine) had suppressed viremia for >12 weeks (post-ART). These patients received peg-interferon alpha 2b two weeks prior to ART initiation. PMBCs were subjected to FACS to enrich activated CD4+ T (CD3+/CD4+/CD38+/HLA-DR+) lymphocytes [25]. Total RNA was isolated and ribosomal RNA (rRNA) was depleted. cDNA libraries were produced with primers for amplification randomly throughout the whole transcriptome. Random hexamers were provided in 50-fold excess over oligo-dT primers enriching cDNA of non-polyadenylated transcripts over steady-state mRNA. The cDNAs were ligated with barcode adapters and sequenced on an Illumina HiSeq2500 platform, as reported in El-Diwany et al. (2017) [25]. Raw Illumina paired-end sequence reads were deposited at the European Nucleotide Archive (ENA) under accession number: SRP068424.

### 2.2. Data Processing

We downloaded the raw data and performed bioinformatics analysis using the resources of Minnesota Supercomputing Institute (Mesabi HPC cluster, 30 May 2021). FastQC was run to generate sequence quality plots for each FASTQ file [26]. The Illumina adapters were removed with Trimmomatic V0.33 [27] and clean reads were processed under the following parameters: LEADING:3, TRAILING:3, SLIDINGWINDOW:4:16, and MINLEN:0. Filtered reads were aligned to the human reference genome hg38, built GRCh38.p13 (accession PRJNA31257) using BWA-MEM aligner [28] with a specified alignment score threshold T = 19. During read mapping by BWA-MEM, reads mapped to either 5’ or 3’ splice sites in a reverse order qualify as backsplicing junctions (BSJ). The BSJs that mapped to paired 5’ and 3’ splice sites are termed paired chiastic clipping signals (PCCs), and these reads were prioritized [29]. CIRI2 was used to identify BSJ characteristic of circRNAs in all samples [29,30]. The CIRI2 algorithm calculates the number of BSJs and identifies the circular ratio (r), where C stands for the number of circular junction counts (previously designated BSJ) and L for linear reads [30]. By default, CIRI2 implements PCC > 2 as the main filtering criteria based on local re-alignment results provided by BWA-MEM. A series of Python scripts filtered the CIRI2 output and identified reads exhibiting ≥3 BSJ and r ≥ 0.03 in all 38 (19 patients in 2 treatment groups) samples. Raw circular counts were imported into R. DESeq2 package [31] was used to assess the frequency of circRNAs that were differentially expressed.

### 2.3. Gene Ontology and miRNA Prediction Algorithms

Panther was used to run the Gene Ontology (GO) enrichment analysis [32] with a significance criterion *p <* 0.05. The database CircAtlas 2.0 annotated circRNAs by gene locus of origin and predicted complementarity with host miRNAs [33], which utilizes targetScan, miRNAsanda, and PITA algorithms for miRNAs prediction parameters [34]. The MicroRNA Target Prediction Database (http://mirdb.org/, last accessed on 8 August 2021) [35] identified mRNAs with selected MREs within the 3’-UTR exhibiting benchmark prediction scores of ≥50 and also a more robust cutoff of ≥80. Gene functions were collected using GeneCards (https://www.genecards.org/, last accessed on 8 June 2021). Cytoscape software (3.9.0) was used to visualize miRNAs–mRNA interaction networks [36]. shinyCircos package for R (last accessed: 31 January 2022) was used to create circular visualization plots [37]. The code used to perform this bioinformatics analysis can be found at Dora Zucko GitHub and the code for the circos plot at Grinde Lab GitHub last accessed: 31 January 2022 

## 3. Results

### 3.1. Data Quality Parameters after Processing

The sensitivity of circRNA detection is dependent on sequencing depth with commercial sequencing providers suggesting >40 million reads per sample. Published RNAseq research has shown that circRNAs can be mined already from 20 million paired-end reads ≥100 bp (selected references [33,38,39,40]). Sequence reads were collected from the PBMC of HIV-1 positive people before and after ART suppression of viremia (SRP068424) that exhibited, on average, 47.8 million paired-end reads of 100 bp per sample [25]. The lowest depth was 20.7 million and the highest depth was 90 million paired-end reads per sample. The RNA submitted for sequencing was not pre-selected for poly(A) RNA species, but was depleted of ribosomal RNA, which is important to increase sequencing depth of circRNAs. The reverse transcription to generate cDNA was initiated randomly throughout the transcriptome by random hexamer primers, because the use of oligo-dT primers to generate cDNA negatively selects circRNA and disqualifies the data set for mining of circRNA profiles. Therefore, the data set was qualified for robust detection of candidate circRNAs.

After trimming the adapters, the average quality score (Q-score) for all reads was 36. Since Q-score ≤ 30 predicts 1 in 1000 base calls is incorrect, any bases below Q30 were discarded in the quality control process. The Q-scores: forward reads, 37, and backward reads, 35, corresponded to a >99.9% accuracy in inferred base calling. Therefore, the Q-score parameter was suitably robust to proceed with the analysis.

Deduplication is a default step in processing raw reads and preparing the data set for downstream analysis. In the patient data set, 77% of all reads qualified as distinct reads, in which no two sequences were identical. The value serves as a measure of library diversity. If a library is diverse, most sequences will occur only once. Duplicates may arise as PCR artefacts during the template amplification step prior to actual sequencing, optical artefacts detected by the machine or can be biological duplicates. The average guanosine cytosine (GC) content for all reads in the data set was 45%. Regions having 50–60% GC content received the highest coverage, while regions with 70–80% or 30–40% GC content exhibited decreased coverage [41]. Thus, GC content can impact the coverage and introduce bias in data interpretation. We concluded the patient data set exhibited sequence content within a suitable range for coverage.

**Figure 1:**
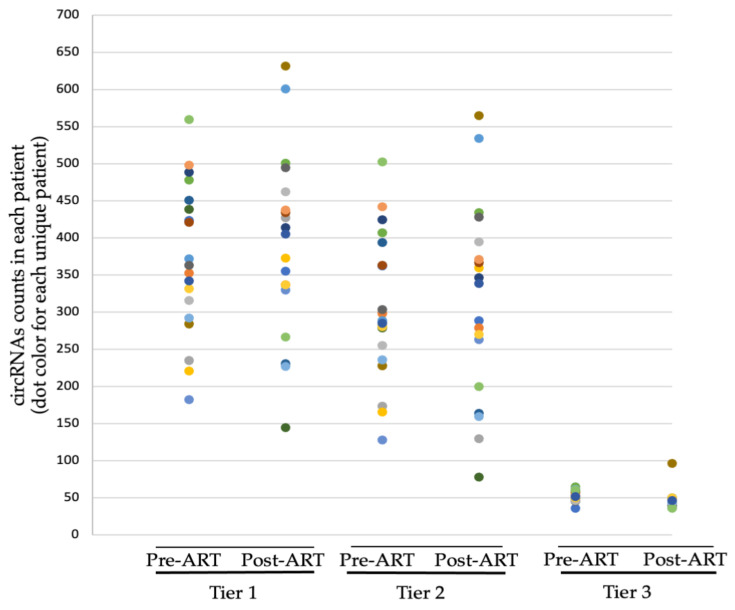
Progressively stringent filtering criteria revealed a similar abundance of patient circRNA pre- and post-ART. Each dot color represents one patient (*n* = 19) per treatment cohort. Bolstered stringency criteria resulted in the~85% reduction in the number of circRNA. Tier 1, Tier 2, and Tier 3 stringency filters. Tier 1 criteria: back-splicing junction count (BSJ) > 1, total number of circRNAs identified across pre-ART was 7053 and 7411 in the post-ART cohort. Tier 2 criteria: BSJ > 3, ratio between circular and linear junction counts *(r)* > 0.03. A total of 5819 circRNAs in the pre-ART and 5973 circRNAs in the post-ART patients. Tier 3 criteria: BSJ > 20, *r* > 0.03. A total of 1014 in pre-ART and 876 circRNAs in post-ART patients.

The CIRI benchmark criteria identified a total of 7053 and 7411 circRNA reads in the pre-ART and post-ART groups, respectively. Next, we applied additional stringency criteria to stratify the data. Tier 1 was the CIRI2 default criteria, PCC > 2, BSJ > 1, which requires at least one BSJ composed of nonlinear 3’ and 5’ splice sites and predicts circRNA are less abundant than linear reads. Tier 2 stringency criteria were developed by Gao et al. and specified BSJ ≥ 3, *r* = ≥0.03 [30]. Tier 2 criteria identified 80% as many circRNA reads across all patients: 5819 pre-ART and 5973 post-ART. As shown in Figure 1, the frequency of circRNAs per patient was similar between Tier 1 and Tier 2. We concluded Tier 2 criteria eliminated low abundance candidate circRNA. Tier 3 stringency criteria specified BSJ ≥ 20, *r* ≥ 0.03 and identified 1014 reads and 876 reads in the pre-ART and post-ART patients, respectively. The Tier 3 criteria enriched high abundance candidate circRNA and significantly reduced the frequency of circRNA. We concluded the collection of circRNA loci identified by the previously established Tier 2 stringency criterion [30] were appropriate for further analysis.

### 3.2. Genomic Origin of circRNAs Suggests Predisposition for Chromosome 17

**Figure 2:**
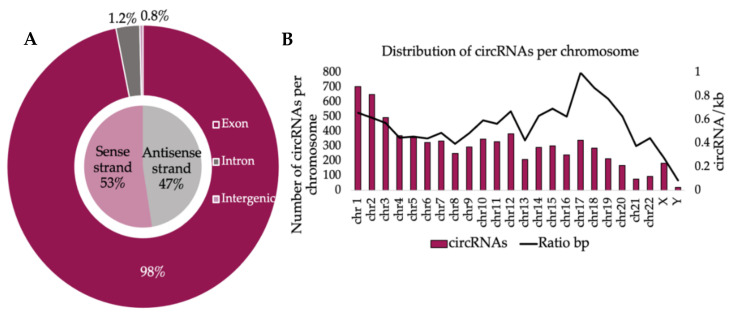
The majority of circRNAs detected in the HIV-1 patient cohort were derived from exons on both DNA strands of chromosomes: (**A**) distribution of circRNA loci corresponding to exon (purple), intron (dark gray), and intergenic sequences (mauve) on the sense (pink) or antisense (light gray) strand; (**B**) number of circRNAs per chromosome (purple bars) compared to circRNA abundance normalized to chromosome size (kb) (black trend line).

Tier 2 circRNAs identified in the pre-ART samples were almost evenly located on the sense and antisense DNA strands (Figure 2A). The vast majority originated from coding sequences within the parental genes (exonic origin) as a result of alternative splicing, while a miniscule percentage was attributed to introns and intergenic regions. Similar trends were identified in the post-ART data set. Our next goal was to identify the genomic distribution of circRNAs and gene of origin.

The circRNAs observed in the pre-ART treatment were most commonly derived from chromosomes 1 (9.5%) and 2 (8.7%), which are the two largest chromosomes in the human genome (Figure 2B, left axis). We then plotted the number of circRNAs originating from a given chromosome normalized to chromosome length (kilobase pairs, kb) (Figure 2B, right axis). The relatively short chromosome 17 (~83,000 kb) produced relatively high numbers of circRNAs compared to, for example, chromosomes 8 and X, which are much larger. Notably, chromosome 17 has previously been identified as predisposed for HIV-1 integration [42]. HIV-1 has been shown to preferentially integrate on chromosome 17 in regions enriched in GC. High gene density (genes/kb) on this chromosome has been considered advantageous for efficient provirus gene expression [43]. Lastly, chromosome 17 is known to be rich in short interspersed nucleotide elements [44], which have been shown to be present on introns flanking the circularized exons [45]. Future measurement of circRNA before and after HIV-1 infection could provide a suitable perspective into a possible role for HIV-1 in promoting circularized exons.

**Figure 3:**
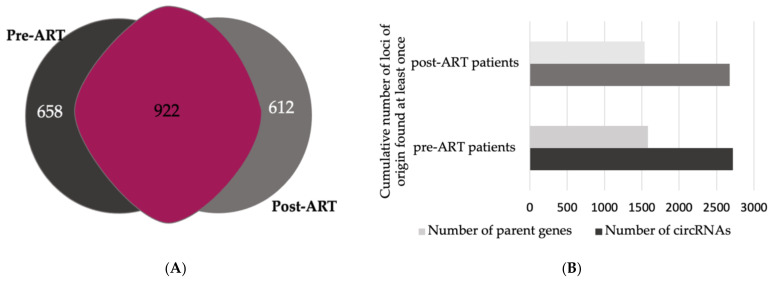

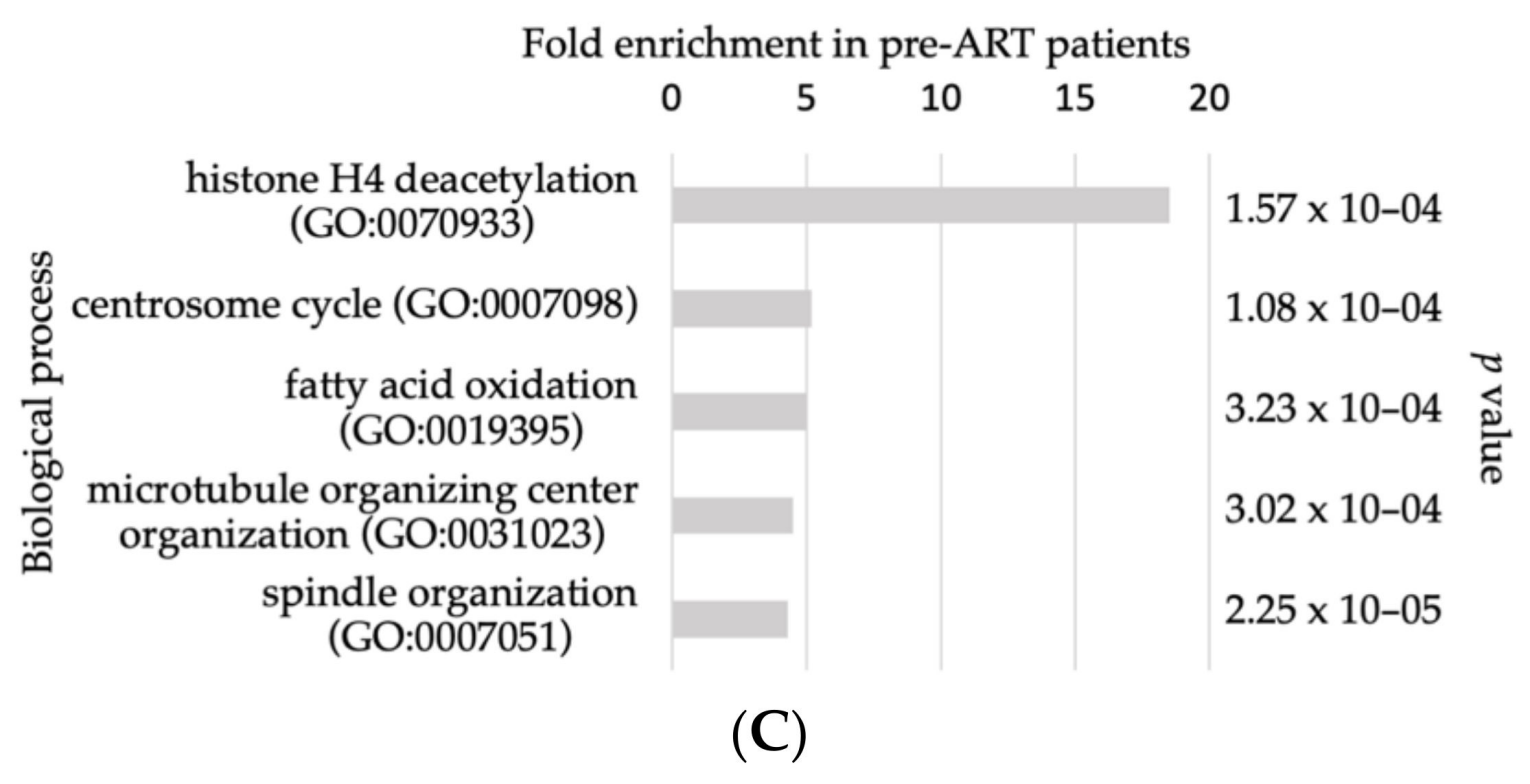
Parental genes gave rise to one or more circRNA indicating alternative circularization. (**A**) Selected parental genes were exclusive to the treatment group. The Venn diagram summarizes unique parent genes observed pre- (darker gray) and post-ART treatment (lighter gray) and parent genes overlapping between the two groups (mauve). (**B**) The number of parent genes (light gray bars) giving rise to circRNAs was consistently lower in pre- vs. post-ART patients when compared to the number of circRNAs (dark gray bars), which is in accordance with published literature. (**C**) Gene ontology enrichment analysis of circRNA loci predicted biological processes of parental genes. The top 5 enriched GO terms (>2 fold change, *p* < 0.05) in pre-ART patients. *p*-values are listed on the right vertical axis.

We then identified the gene of origin for each circRNA in the pre- and post-ART samples, 1581 and 1535 genes, respectively. A Venn diagram depicts that of those genes, 658 genes were specific for pre-ART status, 612 for post-ART status, and 922 genes were in common between both treatment groups (Figure 3A). The cumulative number of circ-RNAs identified at least once was greater than the number of gene loci (Figure 3B). No significant difference in the number of circRNA identified was observed in either the pre-ART or post-ART patients (dark gray bars). The data indicated that one gene can produce multiple circRNA isoforms. These results are consistent with previous reports that a single gene locus may yield more than one circRNA [46,47,48,49]. Differing patterns of circularization favoring flanking exons for circularization have been associated with the competition for RNA nt–nt pairings from alternatively spliced introns within a single RNA [47].

### 3.3. Identification of circRNAs Derived from Genes Associated with HIV-1 Gene Expression

The Gene Ontology Resource was used to perform gene ontology enrichment analysis powered by the Panther Classification System [32]. Fold enrichment represents the background frequency of all genes annotated to that term to the sample frequency of those genes [50]. In the pre-ART group, parental genes of circRNA were significantly enriched for biological processes required for HIV-1 replication (Fisher exact test *p <* 0.05, log-fold change > 2). The most prominent category was “histone deacetylation”, having 18-fold enrichment in the pre-ART group (Figure 3C). Histone deacetylation is one of the best known GO categories attributed to latent HIV-1 in resting CD4+ cells post-ART, when viral loads in the blood are reduced to an undetectable level [51]. The circRNA upregulation was observed for the exonic sequences of RCOR1, HDAC1, HDAC4, and HDAC9. A possible explanation is the competitive biogenesis of circRNA was attributable to alternative splice variants encoding mRNA templates for HDAC protein synthesis [2].

Upon investigating the parental genes in the post-ART patients, the macroautophagy process was the GO term showing the most significant fold enrichment (Fisher exact test *p* < 0.05, log fold change > 2). The circRNA upregulation was observed for ARL8B, ATG2B, ATG7, ATG14, CALCOCO2, LAMP2, MFSD8, STX17, TBC1D5, TECPR1, UFL1, and VPS4A. Autophagy is an intracellular degradative process used for recycling proteins from the cytoplasm to generate macromolecular building blocks and energy under stress conditions, to remove damaged organelles, and to maintain cellular homeostasis by playing a role in both innate and adaptive response to viral infection [52]. It is known that HIV-1, HCV, and other viruses antagonize the antiviral function of autophagy to facilitate the viral life cycle. For instance, HIV-1 Vpr antagonizes autophagy to promote propagation of the virus [53,54,55]. Future studies are warranted to investigate whether the circRNA identified here serve to circumvent autophagy.

The most significant GO terms that were common to pre- and post-ART patients were histone H3-K36 methylation (fold enrichment 14.2, *p* value = 5.3 × 10^−6^) and the corticosteroid receptor signaling pathway (fold enrichment 12.8, *p* value = 8.4 × 10^−4^). Histone methylation is associated with active provirus transcription, implicating the upregulation of these circRNA in the epigenetic regulation of host or viral gene transcription [56]. The agonists of glucocorticoid receptors, such as dexamethasone, have been shown to silence the provirus in human microglial cells [57]. These observed GO categories are representative of the virus-host stand-off in HIV-1-infected patients.

### 3.4. circRNA Profiles Exclusive to HIV-1 Patients’ Viremic Status

After categorizing the circRNA genes of origin, we applied a bifurcated approach to identify circRNA candidates of interest. First, we employed a negative binomial distribution model [31] and identified only one circRNA that was differentially expressed, the previously annotated circRNA hsa-SATB1_AS1_0002, which we refer to as circSATB1-as, was upregulated in pre-ART patients (log2FC = 23, *p* value = 4.4 × 10^−7^). While finding a single significantly upregulated circRNA was unexpected, this observation corresponds to the trends in mRNAs from the same data set. El-Diwany et al. observed that not a single mRNA was differentially expressed pre- vs. post-ART across the 19 individuals in the cohort using a standardized approach for the analysis [25]. The results indicated post-transcriptional regulation of the mRNA expression would be paramount to manifest changes in gene expression.

The circSATB1-as is an 897 bp exonic sense-strand circRNA located on human chromosome 3 that originates from the gene encoding SATB1 long non-coding RNA. The antisense strand of the parental gene encodes SATB1 protein (special AT-rich sequence-binding protein 1) that plays a key role in T-cell development, and it is responsible for the maintenance of chromatin architecture, providing a specific binding site for chromatin-remodeling enzymes such as HDACs. Interaction between Tat and SATB1 has been investigated in HIV-1-infected T cells and indicate that Tat competitively displaces SATB1-bound HDAC1 thus increasing the acetylation of the promoter [58]. SATB1 was found to specifically bind to HIV-1 integration sequences within the host genome, and upon knock down, HIV-1 integration at SATB-1 binding regions was disfavored [59]. Future experiments are warranted to determine if circSATB1-as is a biomarker of productively infected cells that are directly or indirectly downregulated by ART.

The second approach to evaluating the data set identified the prevalence of circRNAs in the patient population pre- or post-ART treatment. The unique circRNAs identified in the patient cohort are summarized in Table 1. As expected, fewer unique circRNA were prevalent in all of the patients than subsets of the cohort. 

Seven circRNA were common to all viremic patients and these seven were not detectable in all post-ART-patients (Table 1). Those candidates were: circANKRD17 (hsa-ANKRD17_0008), hsa-ATXN1_0001 (circATXN1), hsa-FAM13B_0019 (circFAM13B), hsa-FBXW7_0005 (circFBXW7), hsa-HIPK3_0001 (circHIPK3), hsa-PHC3_0020 (circPHC3), and hsa-ZNF609_0001 (circZNF609). We investigated the literature for connections between the seven circRNA loci and viral infection.

The upregulation of parental gene ATXN1 has been shown in CD8+ cells of HIV-1 positive patients [60]. ANKRD17 was found to be an HIV-1 Vpr-associated DNA repair protein [61] and to promote viral replication in genome-wide screens of host dependency factors by interacting with HIV-1 Vpr [62,63]. Thus, we considered the upregulation of circANKRD17 could diminish viral replication through post-transcriptional downregulation of ANKRD17. For the five other parental gene loci, we could not identify published roles related to HIV-1.

The seven circRNA varied in mature length from 620 (i.e., circFBXW7) to 1634 bp (i.e., circANKRD17) with the length of circATXN1 being undocumented. With the exception of circHIPK3, which is reported to be highly expressed in skeletal muscle, these circRNAs are abundantly expressed in the spleen, a major reservoir of CD4+ T-cellsand latently infected HIV-1 during ART [64]. Genome scale analysis across rodents and other mammals documented GC content is a reliable prognostic factor for hotspots of circRNA biogenesis [65]. A GC content of <52% when compared to non-circRNA producing genes is a feature common to genes producing circRNAs. The genes encoding the circRNAs (ANKRD17, ATXN1, FAM13B, FBXW7, HIPK3, PHC3, SATB1-a, and ZNF609) exhibited a relatively low GC content (37–50%, respectively) in agreement with this prediction for circRNA loci. We concluded the circRNA detected in all 19 (100%) patients’ blood pre-ART were robust candidates to investigate circRNA–miRNA–mRNA networks influencing viral replication.

### 3.5. Identification of MRE in the Host circRNAs Upregulated in Viremic Patients

One of the most widely investigated activities of circRNAs is serving as an MRE that sequester miRNAs to regulate post-transcriptional gene expression [4,5,6,7]. We predicted MREs in the circRNA cohort using the circAtlas web tool. MREs were not detectable in three circRNAs: circATXN1, circFAM13B, and circFBXW7, while a collection of 16 MREs were detected in five circRNAs and some MREs were redundant between the circRNAs (Table 2). For instance, two circRNA contain MRE complementary to hsa-miRNA-141-5p and hsa-miRNA-197-3p.

**Figure 4:**
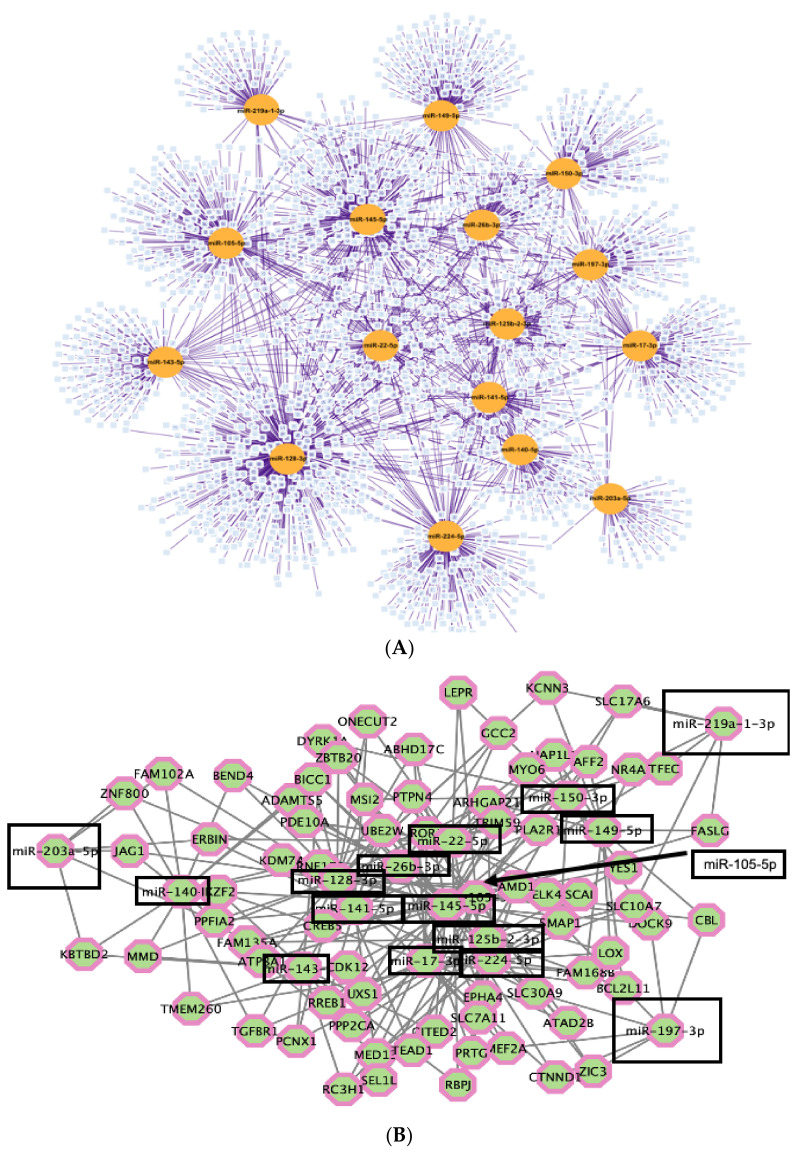

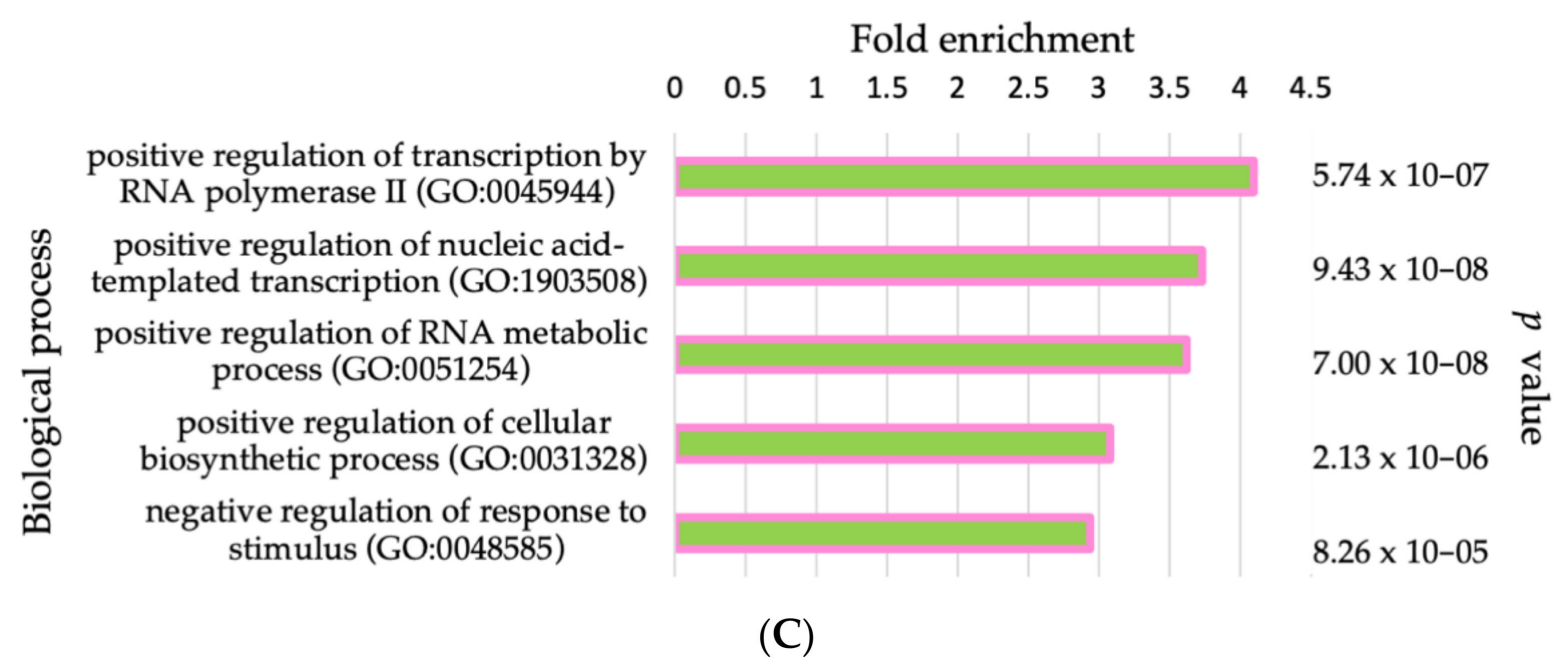
Competing circRNA–miRNA–mRNA network analysis identified a connection between the circRNAs exclusive to all 19 viremic patients and post-transcriptional control of genes governing transcription of host and pro-viral RNAs. (**A**) The miRNAs predicted to competitively target circRNA–MRE and host mRNA–MRE. Orange circles, MRE cognate miRNA from Table 2 exhibited complementarity to candidate circRNA. Light blue squares, mRNA that had an MRE targeted by the indicated miRNA. Purple lines, connections between the nodes. (**B**) Enhanced stringency network: mRNAs containing at least 3 MREs from (**A**). Black box, MRE cognate miRNA from Table 2; Octagons, host mRNA from (**A**) that were targeted by three or more cognate miRNAs in common with circRNA. (**C**) Top GO terms of mRNA identified in the enhanced stringency network in (**B**). *p*-values are listed on the right vertical axis.

Next, MREs that are shared between circRNAs, HIV-1 RNAs, and host RNAs were predicted by miRDB. Competing interaction for the cognate miRNAs was analyzed between the circRNA, host mRNA and viral mRNA by using Cytoscape. The network analysis identified MREs in common between 2184 host mRNAs and the candidate circRNA (Figure 4A, Supplementary Table S1). To increase positive predictive power for actionable genetic screening, we filtered the results to identify the host RNAs containing three or more of the candidate MREs. The increased stringency eliminated 2114 genes and provided 70 genes of particular interest (Figure 4B, Supplementary Table S2). Annotated functions of these genes were identified and each of the top five categories were biological processes related to gene expression and RNA metabolism. The most enriched GO category was positive regulation of transcription by RNA polymerase II (Pol II) (FC = 4.3, *p* = 5.74 × 10^−7^) (Figure 4C). The genes enriched in the 16 MREs were: ATAD2B, CDK12, CITED2, CREB5, DYRK1A, ELK4, JAG1, KDM7A, MYO6, MED13, MEF2A, NR4A3, ONECUT2, RBPJ, RREB1, SLC30A9, TEAD1, TFEC, TGFBR1, YES1, and ZIC3. The observed competing endogenous network was exclusive to the viremic patients. The results posit the hypothesis that MREs in the circRNAs binding cognate miRNAs competitively downregulate RNA silencing of cognate mRNAs and upregulate their protein products to bolster viral replication.

### 3.6. Six MREs Were in Common between HIV and the Host circRNAs and mRNAs

**Figure 5:**
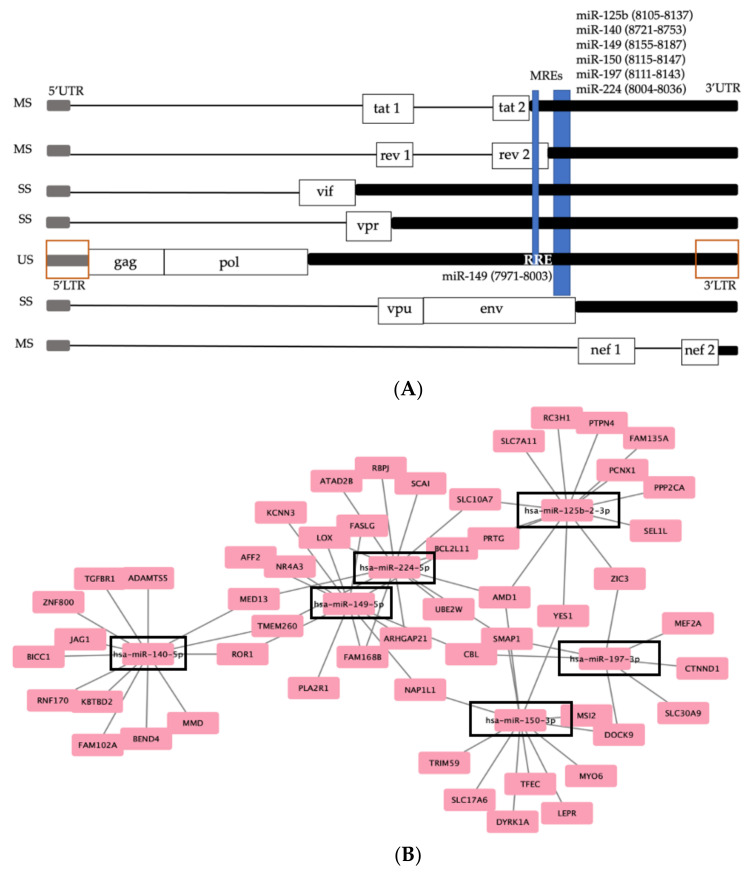
CircRNAs contain MREs previously identified in the HIV-1 3’-untranslated region (UTR). (**A**) HIV-1 precursor RNA is processed into 3 categories of mRNA: completely spliced, MS; incompletely spliced, SS; unspliced, US. Grey rectangles, the 5’-UTR; black rectangles, 3’ UTRs; labeled boxes, open reading frames;thin lines, excised introns;blue vertical lines, MREs designated by cognate miRNA; MRE coordinates; orange box, position of 5’ and 3’ long terminal repeats (LTR) in the precursor RNA to all mRNAs; RRE, Rev responsive element. (**B**) The interaction network posits the six MRE in HIV-1 (**A**) are incommon with the indicated host mRNAs (pink boxes). Each gene locus exhibited 3 or more copies of the candidate MRE for each cognate host miRNA (black outline). Names of genes are listed Table S3.

Of the MREs in common between circRNA and host mRNA, only six were also in common with HIV-1 RNA [66]. Using the NCBI BLAST tool and aligning cognate miRNAs sequences (32 nt) to an HIV-1 reference genome (accession AF033819), we identified the 6 MRE are within the 3’-UTR of tat, rev, vif, vpr, and the gag-pol mRNAs, but not env or nef mRNAs (Figure 5A). Five of the MREs were present once. The MRE targeted by miRNAs-149 was present twice in the 3’-UTR of the completely spliced tat mRNA, and in the incompletely spliced vif and vpr mRNAs and the unspliced RNA [66], most notably within the Rev responsive element (RRE) that requires binding of Rev for production of virion components. We assessed MRE in the candidate circRNA may coordinately affect HCV activity in co-infected patients. Literature searches identified 13 out of 16 total MREs to be involved in both HIV-1 and other virus infections (Table 2).

We then stratified the host genes containing the six MREs in common with HIV-1 and identified 51 host genes (Figure 5B). There was no significant enrichment for any GO category, hence we concluded these miRNAs likely affect the virus, rather than display importance for the virus–host interface. By comparison, the residual MRE (*n* = 10) observed exclusively in host mRNAs identified a network of 70 genes (Supplementary Materials Table S3). The most enriched GO category was again “positive regulation of transcription by RNA Pol II” (FC = 4.1, *p* = 5.74 × 10^−7^), and the remaining top four were the same biological processes observed in Figure 4C. This similarity was expected based on the fact that GO enrichment analysis yielded no significant results for biological processes of mRNAs sharing MREs with the virus. We concluded that the competitive interference of RNA silencing by circRNAs has the capacity to manipulate host miRNAs to upregulate virion proliferation in pre-ART patients.

**Figure 6:**
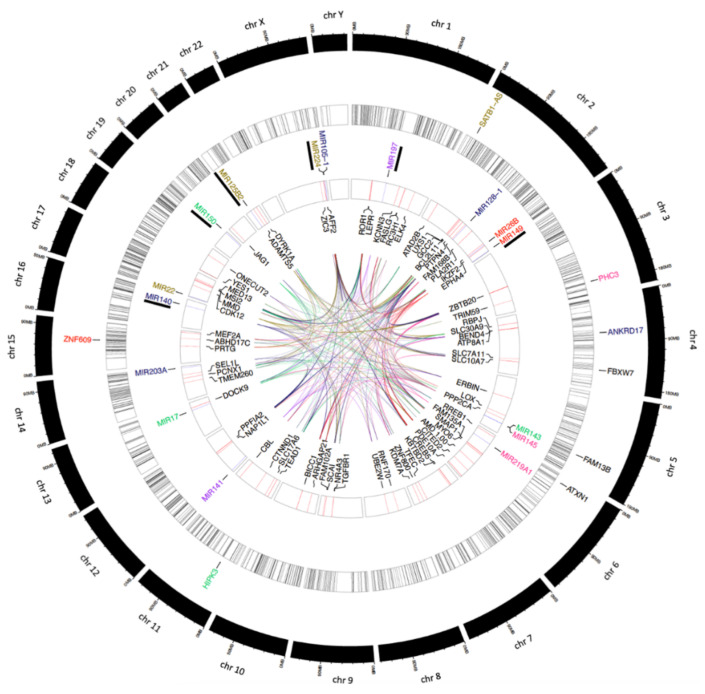
Static view of the Interactive Circos tool that visualized the predicted competing endogenous network of circRNA-miRNA-mRNA interactions originating from seven circRNA singled out in viremic versus non-viremic patients. From outside in: layer 1 (black rectangles)—human chromosomes; layer 2 (colorful text)—high-stringency circRNAs present in all 19 viremic patients (*n* = 8), designation of parental gene IDs and genomic location; layer 3 (black bars in white rectangles)—tier 2 circRNAs (*n* = 2720) in pre-ART cohort. The thickness of the bars was determined by the size of circRNAs based on their genomic start and end coordinates; layer 4 (colorful text)—miRNAs (*n* = 16) targeted by circRNAs from layer 2. Black underline signifies miRNAs targeting the HIV-1 genome. Purple, two miRNAs that are dually targeted by circPHC3 and circANKRD17; layer 5 (red and blue bars in white rectangles)—red bars signify mRNAs (*n* = 70) targeted by at least 3 miRNAs (blue bars) from layer 4. The thickness of the bars was determined by the size of mRNA based on their genomic start and end coordinates; layer 6 (black text)—names of mRNAs from layer 5; layer 7 (colorful ribbons)–the ribbons mark the interactions between miRNAs and target mRNAs. Ribbons are color-coded based on the colors in layer 2 and layer 4, e.g., red-colored text marks that circZNF609 interacted with miRNA26b and miRNA 149. Red ribbons, miRNA26b targeted PDE10A, CDK12, BICC1, MYO6, MEF2A, GCC2, CREB5, PRTG, TRIM59, KDM7A, and BEND4; miRNA149 targeted FASLG, ARHGAP21, NR4A3, FAM168B, NAP1L1, PLA2R1, LOX, ROR1, BCL2L11, KCNN3, CBL and AFF2.

To integrate the trends observed in this *in-silico* analysis, we visualized the overlapping interactions between the host gene loci of the circRNA, the MRE of the circRNA in common with host mRNA and HIV-1 mRNAs, and the collaborative interaction between these host components (Figure 6). While the circos plot provides a high-level view of the potential relationship between the seven circRNA singled out in viremic versus non-viremic patients, miRNAs, and host gene linear RNAs, we developed a web-interface Interactive Circos (last accessed on 31 January 2022) to trace individual components of the intricate post-transcriptional network. To the best of our knowledge, this is the first characterization of circRNA competing endogenous RNA network in pre-ART viremic HIV-1 patients. The results provide detailed information to guide future validation studies.

## 4. Discussion

By repurposing published RNAseq data from the ENA public read repository, we tested parameters to detect circRNA profiles in a robust clinical study by El-Diwany et al. that measured transcripts in patients’ blood during HIV-1 viremia and after successful suppression by ART [25]. By identifying over 14,000 circRNA reads from patients in both groups and characterizing the differences in circRNA profiles between the viremic and ART-treated, we confirmed the hypothesis that circRNAs can be detected in previously published RNAseq data and statistically significant differences between cohorts were detectable.

Despite significant progress in the last several decades in the development of ART, pre-exposure prophylaxis for at-risk groups, and preventative vaccine prototypes, the HIV-1–host interface remains inadequately defined. The circRNA identified exclusive to all 19 patients pre-ART generated circRNA–miRNA–mRNA networks that have the potential to promote viral replication. The GO category that was enriched by the circRNA loci in all viremic patients was RNAPII (fold enrichment > 4, *p* = 5.7 × 10^−7^). Upon integration into the host genome, RNAPII is required for transcription of the provirus. HIV-1 has been shown to manipulate the activity of RNAPII intricately through the regulatory protein Tat bound to the trans-activation response (TAR) element [67]. Tat bound to TAR on the nascent viral RNA trans-activates the host positive transcription elongation factor b to release RNAPII for the elongation phase of Pol II activity, which together with Rev/RRE is essential to manifest productive viral gene expression and virion proliferation [68]. The importance of HIV-1 manipulation of Pol II activity to viral replication is underscored: more than half of the genes belonging to the enriched RNAPII GO category are annotated in HIV-1 human interaction database [69,70,71].

We generated an accessible network analysis tool Interactive Circos to efficiently trace directed interactions for each circRNA and pairs of miRNA-cognate target mRNA. The constructed competing circRNA–miRNA–mRNA network predicts circRNA sequester miRNAs whose activity diminishes HIV-1 replication (Table 2). Both miRNAs-150 and miRNAs-125b were found to be downregulated in HIV-1-infected PBMC from patients (miRNAs-150) and CEMx174 cells (miRNAs-125b) [22]. Quantitative PCR (qPCR) verified that diminished miRNA-125b enhances viral replication [72], positing the testable hypothesis circRNAs physically squelch these miRNA and recapitulate enhanced replication. Previously, both miRNA-150 and miRNA-125b have shown an increase in resting primary CD4^+^ T cells compared to activated CD4^+^ T cells productively infected with HIV-1 [66]. The differences in circRNA, miRNA, or mRNA levels before and after infection were not attainable in our study. Instead, our study ascertained RNA levels during patients’ viremia and after ART suppression of viremia.

With resting T cells being the main reservoir of latent infection, the potential downregulation of circRNA squelching these miRNAs conceivably contributes to latency in these cell populations. After inhibiting these miRNAs with antisense inhibitors in resting CD4+ T cells, authors reported >10 times more HIV-1 particles were generated than from cells treated with negative control inhibitor, verifying miRNA inhibition activates latent virus [66]. Our findings in this study predict upregulated circSATB1 and circHIPK3 indicative of viremic state may function as RNA silencing suppressors by sequestering circulating miRNAs-125b and miRNAs-150 from carrying out interactions that upregulate HIV-1 propagation. This hypothesis requires validation, especially since the differential expression analysis by El-Diwany failed to detect significant change in the steady state mRNA transcriptome pre- and post-ART in the data that were reanalyzed in this report [25].

Additional cognate miRNA predicted in this study (Table 2) were detected in large scale screens [63,70] and experimental validation determined miRNAs-149 impairs viral replication in Jurkat and MT4 human T-cell lines by binding MREs within the intronic gag sequences and the distal 3’-UTR [73]. Notably, a qPCR screen identified miRNAs-197 in extracellular vesicles (EVs) compared to CD4+ T cells isolated from plasma of ART-naive HIV-1-positive individuals [74]. EVs, comprising exosomes and microvesicles, are circulating carriers of bioactive molecules and miRNA among cells mediating viral spread. We have not identified experimental studies assessing miRNAs-140 and miRNAs-224 impact on HIV-1 replication. However, overexpression of miRNAs-140 was reported to inhibit both classical swine fever virus by targeting host factor RAB25 and mink enteritis virus replication by repression of its receptor, verified by qPCR and measurements of luciferase activity in cell cultures [75,76]. Similarly, upregulation of miRNAs-224 was found to suppress replication of hepatitis B virus in hepatic cells measured by antigen ELISA [77]. It is unknown if these miRNAs target either of the viral genomes. In closing, the observations made in this study support the hypothetical role of circRNAs as nucleic acid-based RNA silencing suppressors. The combined downregulation of several circRNAs may be necessary to detect circRNA activity on viral fitness. CircRNAs specific for viremic patients warrant investigation as candidates for targeted anti-HIV therapy.

## 5. Limitations

The limitation of this in silico analysis is that experiments are warranted to validate the predictions. The top priority is quantifying the levels of the seven top candidates in the patients’ primary T cells, followed by verifying the corresponding candidate proteins’ increase in response to the downregulation of the candidate circRNA. Second, transcriptomics data were not available on the patient samples prior to HIV-1 and HCV co-infection, and HCV replication in the patients’ hepatocytes was not documented either. Predicted in the networks was the ONECUT2 transcription factor that is known to be active in hepatic cells. ONECUT was predicted in the network encompassing circANKRD17–miRNA141-5p, circPHC3–miRNA141-5p, and circSATB1–miRNA-22-5p, but involvement of mir122 was not detected. In closing, host circRNA profiles in viremic patients changed significantly after suppression by ART. Future investigation are warranted to define whether circRNA biomarkers of HIV-1 viremia will be viable therapeutic targets to prevent AIDS. The competing endogenous network pipeline developed in this study and displayed by an Interactive circos plot, visualizes the interplay between individual circRNA–miRNA–target mRNA important for HIV-1 and other potentially other diseases.

## Figures and Tables

**Table 1:** High stringency Tier 2 criteria resolves circRNAs exclusive to viremic patients.

Members of Cohort		Prevalence of Unique circRNAs	
Proportion	Abundance	Pre-ART ^a^	Post-ART
100%	19	7	0
75%	14	65	61
50%	9	147	167
25%	5	339	357
15%	3	588	638
5%	1	2720	2675

^a^ ART, antiretroviral therapy.

**Table 2:** Competing RNA interactors are proposed by network analysis of candidate circRNA exclusive to 19 viremic patients.

Designation Role of Parental Gene Locus	MREs Identified in Host circRNA	MRE Identified in HIV-1 3’UTR	Anti-HIV-1 Activity of Cognate miRNA	Frequency of Host mRNAs with MREs	Other Virus-Related Activity of Cognate miRNA
circANKRD17 Upregulates dsRNA receptor DDX58 and IFIH1 signaling pathways in antiviral innate immune response	hsa-miR-105-5p	Not identified	Not identified	231	Not identified
	hsa-miR-128-3p	Not identified	Not identified	464	Herpesvirus 1, Influenza A virus, Murine gammaherpesvirus 68, Respiratory syncytial virus
	hsa-miR-140-5p	Yes	Not identified	91	Classical swine fever virus, Mink enteritis virus
	hsa-miR-141-5p	Not identified	Not identified	101	Enteroviruses, Epstein–Barr virus
	hsa-miR-197-3p	Yes	Maybe	106	Enteroviruses, Hepatitis B virus, SARS-CoV2
	hsa-miR-203a-5p	Not identified	Not identified	61	Hepatitis C virus, Influenza A virus, Sendai virus
circHIPK3 Regulates transcription and apoptotic pathways	hsa-miR-17-3p	Not identified	Not identified	146	Not identified
	hsa-miR-143-5p	Not identified	Not identified	133	Influenza A virus
	hsa-miR-150-3p	Yes	Yes	132	Dengue virus, SARS-CoV2
circPHC3 Maintains repressed state of genes through chromatin remodeling and histone modification	hsa-miR-141-5p	Not identified	Yes	101	Enteroviruses, Epstein–Barr virus
	hsa-miR-145-5p	Not identified	Not identified	311	Hepatitis C virus, Zika virus, SARS-CoV2
	hsa-miR-197-3p	Yes	Maybe	106	Enteroviruses, Hepatitis B virus, SARS-CoV2
	hsa-miR-219-a-1-3p	Not identified	Not identified	66	Avian Influenza virus, SARS-CoV2
circSATB1 Regulates chromatin structure and remodeling	hsa-miR-22-5p	Not identified	Not identified	150	Not identified
	hsa-miR-125b-2-3p	Yes	Yes	141	Hepatitis C virus, Japanese encephalitis virus
	hsa-miR-224-5p	Yes	Not identified	183	Hepatitis B virus
circZNF609 Binds promoters with paused Pol II	hsa-miR-26b-3p	Not identified	Not identified	106	Newcastle disease virus, Sendai virus, Vesicular stomatitis virus
	hsa-miR-149-5p	Yes, twice	Yes	155	Hantaan virus, Influenza A virus
circATXN1 circFAM13B circFBXW7	None identified	N/A	N/A	N/A	N/A

## Data Availability

The code used for this bioinformatics analysis is available at https://github.com/zucko001/circRNA-in-HIV-1-patients and https://github.com/GrindeLab/circRNA, last accessed on 31 January 2022. Interactive version of the circos plot can be found at https://kblcircosgraph.shinyapps.io/circos/, last accessed on 31 January 2022. Other data are available upon request.

## Supplementary Materials

The following supporting information can be downloaded at: https://www.mdpi.com/article/10.3390/v14040683/s1, Table S1: The list of miRNAs and their target genes from Figure 4A (representing all miRNA-mRNAs interactions); Table S2: the list of miRNAs and their target genes from Figure 4B (representing mRNAs targeted by at least 3 miRNAs from Figure 4A). Table S3: the list of miRNAs observed exclusively in the host and the corresponding mRNA targets.
